# Differential partitioning of seed-inhabiting methylotrophs in the endosphere of wheat plants

**DOI:** 10.1186/s12915-025-02473-8

**Published:** 2025-12-12

**Authors:** Apekcha Bajpai, Amit Kumar Dash, Bharati Kollah, Rakesh Parmar, M. H. Devi, Ethan Rodrigues, Françoise Bringel, Santosh Ranjan Mohanty

**Affiliations:** 1 ICAR-Indian Institute of Soil Science, Bhopal, Nabibagh 462038 India; 2 ICAR-Central Rice Research Institute, Cuttack, Odisha 753006 India; 3 Génétique Moléculaire Génomique Microbiologie, UMR 7156, Université de Strasbourg / Centre National de La Recherche Scientifique (CNRS), Strasbourg, 67000 France

**Keywords:** Wheat, Endophyte, Endosphere, Seed, Pink-pigmented facultative methylotroph, Root-to-shoot peganine gradient

## Abstract

**Background:**

Both endophytes, microorganisms that reside within plants, and methylotrophs, which grow using methanol produced from plant leaves, play key roles in protecting plants against biotic and abiotic stresses. However, the source of endophytes and the mechanisms of their selection in plants are poorly understood. Therefore, experiments were carried out to identify wheat seed methylotrophic endophytes and evaluate their partitioning in root, stem and leaf of aseptic-controlled plants cultivated from surface-sterilized seeds.

**Results:**

The counts of endophytic methanol utilizers were higher in leaf tissue than in stem, root and seed, as estimated using viable counts and qPCR targeting *rrn* gene. The methanol dehydrogenase subunit *mxaF* gene was PCR-detected in all pink-coloured isolates that grew using methanol or succinate. These pink-pigmented facultative methylotrophs (PPFM) were dominant in shoot tissue. Using mass spectrometry for alkaloid content analysis, peganine was detected as a peak 16.6% higher in root than shoot. Root extracts and peganine alone inhibited the growth of PPFM.

**Conclusions:**

PPFM transmitted from seed are more abundant in shoot than root. How plant compounds such as peganine are involved in the methylotrophic endo-phytomicrobiome dynamics remains to be better characterized.

**Supplementary Information:**

The online version contains supplementary material available at 10.1186/s12915-025-02473-8.

## Background

Vascular plants and microbes together have coevolved and inhabit nearly all plant parts, forming an interdependent ‘holobiont’ that improves the fitness of plants [1, 2]. Endophytes are found in both vegetative and reproductive inner parts of many plant species [3–5]. The presence of the endophytic community confers increased plant resistance to biotic or abiotic stresses, breaks seed dormancy, enables germination, promotes plant growth [6, 7] and favours heavy metal tolerance and bioaccumulation [8]. Bacteria present in plant seeds are endophytes that can be transmitted to other plant tissues, persist throughout the lifecycle of plants and be transferred vertically from one generation to the next [4, 9]. Endophytes can sometimes also cross the stomata and switch from endophyte to epiphyte lifestyle [10]. A core microbiome of bacterial endophytes (shared subset of bacterial taxa found in the seed endosphere of several plants) can be transferred from seed to the germinating plant [11]. Nonetheless, this transfer process is still poorly understood and may involve selective processes determined by plant tissues [12, 13] and ecological processes involved in seed-to-seedling transmission of bacteria [14, 15]. Humidity and solar radiation are two such environmental variables that determine both endophytic and epiphytic microbiota of the phyllosphere [16].

Seed microbiome studies of plant species revealed the occurrence of phyla belonging to Actinobacteria, Firmicutes and Gammaproteobacteria, including Pseudomonadales and Enterobacteriales [5, 17, 18], but with low species richness [11, 12, 19, 20]. Distribution and abundance of *Pantoea* sp. in wheat seedlings have been studied in detail, but other taxa have been overlooked [4]. In particular, the Alphaproteobacteria *Methylobacterium* is one such monophyletic group that resides in the seed and colonizes the plant in the early phase of germination [21, 22]. These bacteria have plant growth-promoting attributes and protect plants from abiotic stress factors [23–25]. Methylotrophs use reduced C1 compounds as a source of carbon and energy, including methanol produced by plant leaves [26]. Facultative methylotrophs can also utilize multicarbon compounds, in addition to reduced C1 compounds. Among facultative methylotrophs, *Methylobacterium* that form pink-coloured colonies are called pink-pigmented facultative methylotrophs (PPFM). In the phyllosphere, PPFM encounter competitive and cooperative interactions with heterotroph residents. Niche-overlap index, which measures the similarity in carbon source utilization between strains, inversely correlates with their ability to coexist. This gives a unique advantage to methylotrophs as they assimilate methanol emitted by plants [27]. Antibiotics-resistant strains of PPFM inoculated onto red perilla seeds were found to be vertically transmitted from seeds to leaves, but some strains were more successful colonizers than others [28]. PPFM are major inhabitants of the phyllosphere, yet their endophytic mechanism for distribution within wheat plants is poorly understood, notably their role in improving plant health [27, 29].

In this context, the present study was undertaken to characterize the diversity of endophyte methylotrophs in wheat seed and to reveal how PPFM are partitioned between the root and the shoot systems. The hypothesis of our study is that the PPFM are selectively distributed from seeds to various parts of the plant to offer ecological advantages to the plant. The key questions were as follows:

(i) Do wheat seeds harbour bacterial diversity including PPFM?
(ii) Is the transmission of PPFM from seed to root and shoot selectively partitioned?
(iii) How does the wheat plant favour the distribution of the methylotrophic community within its plant tissue?

**Fig. 1:**
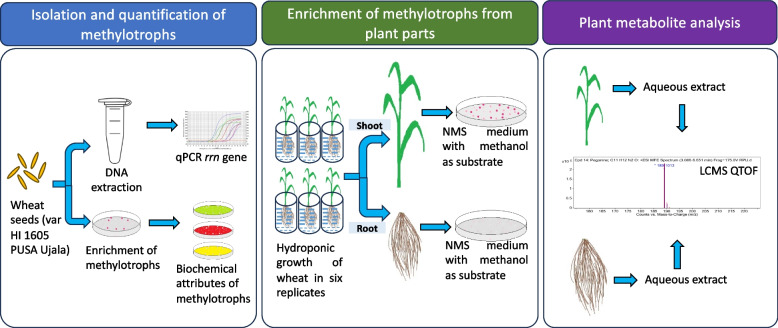
Outline of the experiments carried out to study the differential partitioning of seed inhabiting methylotrophs in different wheat plant parts. The experiment was undertaken in three phases: Methylotrophs abundance and their biochemical attributes were evaluated (first phase, left panel). Methylotrophs were enriched from root and shoot using NMS medium with methanol as substrate to evaluate their population dynamics (second phase, middle panel). Metabolites were identified through LC–MS QToF to determine the mechanism of plant-methylotrophs interaction in different plant parts (third phase, right panel)

To answer these questions, three experimental phases aimed to characterize the taxonomic bacterial diversity in wheat seed and their distribution in different plant parts, as outlined in Fig. 1. First, the abundance of eubacteria in wheat seed was evaluated along with their biochemical attributes. In the second phase, surface-sterilized seeds were germinated and grown under the controlled environment of a plant growth chamber. PPFM were isolated from seed, root and shoot parts and were evaluated based on methanol utilization and biochemical tests. In the third phase, metabolites from different plant parts were identified to explore potential mechanisms of selective endosphere colonization of PPFM in different plant parts of the wheat plant.

## Methods

### Wheat variety and epiphyte seed sterilization

One gram of wheat seeds (variety HI 1605 Pusa Ujala; Additional file 1: Table S1) procured from local market was surface-sterilized with 2% sodium hypochlorite for 2 min and once with 90% ethanol for 1 min followed by 5-min washing with sterile distilled water, as previously described [18]. To assess the efficacy of surface disinfection protocol, the surface-sterilized seeds were tested by placing seeds and monitoring for growth on nutrient agar plates. To evaluate endospheric bacteria, 1 g of surface-sterilized seeds was crushed in a tissue homogenizer (Mini-Beadbeater, Biospec, USA) at 400 rpm for 5 min in sterile PBS buffer (NaCl 137 mM, KCl 2.7 mM, Na_2_HPO_4_ 10 mM and KH_2_PO_4_ 1.8 mM at pH 7).

### Chamber-controlled growth of wheat

**Fig. 2:**
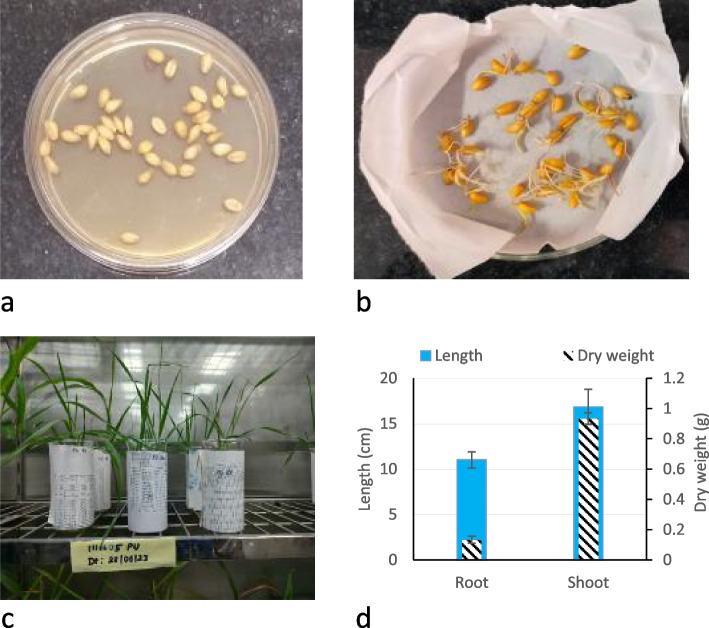
Germination and growth of wheat plants. **a** Surface-sterilized wheat seeds were incubated in nutrient agar plate to test for surface sterilization. **b** Surface-sterilized seeds all germinated on Petri plates. **c** Growth of the germinated seeds resulted in healthy plants after 20 days in ½ MS medium. **d** After 20 days of sowing, root and shoot growth parameters, length (in blue) and dry weight (in hatched black and white) were estimated following standard agronomic measurement. For three replicated observations, arithmetic mean is presented with error bar (standard deviation, significance difference *p* < *0.05* level)

Surface-sterilized seeds (1 g) were germinated in a Petri plate containing sterile filter paper soaked with autoclaved distilled water. Plates were incubated for 1 day in the dark at 25 ± 3 °C in a BOD incubator (Metrex Scientific Instrument, New Delhi). Six germinated wheat seeds were grown in a 200-mL beaker containing MS medium (1/2 × concentration, pH 5.6, HiMedia Inc., Mumbai, India) [30]. The beaker had sterilized floating foam with a hole in the centre and one germinated seed placed carefully per replicate, allowing roots to grow downwards through the hole. Beakers were covered with paper externally to maintain dark conditions for growing roots and kept in a walk-in plant growth chamber (Genesys, India). The plant growth chamber conditions were monitored with controlled temperature (26 ± 2 °C), relative humidity (60%), CO_2_ (400 ppm) and the alternance of light (intensity of 150 μmol m^−2^ s^−1^) for 16 h and darkness for 8 h, for a total duration of 21 days (Fig. 2). After 3 weeks, plants (height—16 cm, dry weight—0.9 g and number of leaves 4) were taken out from the growth chamber.

### Isolation and phenotyping of methylotrophic endophytes

Plant parts (leaf, stem and root) were surface-sterilized using 90% ethanol for 30 s and cut into small pieces. About 1 g of fresh plant tissue material was aseptically transferred to 2 mL of sterile Eppendorf tube containing sterilized glass beads (diameter — 2 mm, HiMedia Inc., Mumbai, India) and mechanically crushed in the presence of 1-mL phosphate-buffered saline in a tissue homogenizer (Mini-Beadbeater, Biospec, USA) at 400 rpm for 5 min [18]. After centrifugation (10,000 g for 5 min), 100 µL of supernatant was spread at the surface of nitrate mineral salts (NMS) agar plates supplemented with 0.5% of methanol and an antifungal filter-sterilized solution (100 mg.L^−1^ of cycloheximide in water) to estimate the abundance of cultivable methylotrophs in the sample [25]. Control plates contained the filtrate of different plant parts without crushing. After 1-week of incubation at 30 °C, on the basis of colour aspects, colonies were re-isolated on new plates, and purified isolates were further characterized. Phosphate solubilization was assessed using growth and zone formation in Pikovskaya’s medium [31], nitrogen fixation using growth in Jensen’s medium [32] and amylase production using colouration in starch hydrolysis agar medium, as described previously [33].

### C1 compound utilization and methanol dehydrogenase-encoding *mxa* gene PCR detection

C1 compound utilization was tested on agar plates of NMS for methanol (1.4 mM, 100 mL), in gas-tight glass chambers (anaerobic culture jar, HiMedia Inc., Mumbai, India, 3.5 L). Purity of the isolates was verified on R2A plates or NMS supplemented with succinate (10 mM). Growth was estimated after 3 days of incubation at 30 °C compared to a control plate incubation without any addition of carbon source. Optical density at 600 nm was measured to assess the ability to utilize carbon sources for growth in NMS liquid media with or without supplementation of either methanol (40 mM) or succinate (10 mM).

Detection of methanol dehydrogenase-encoding *mxa* gene was assessed using PCR. Amplification was carried out in 25 µL of reaction mixture containing 1 × GoTaq (Promega) and 20-pmol primer (mxaF1003, 5′-GCG GCA CCA ACT GGG GCT GGT-3′ and mxaR1561, 5′’-GGG CAG CAT GAA GGG CTC CC-3′) using fragments of isolated colony for DNA template [34]. The PCR program was as follows: initial denaturation at 94 °C for 5 min, followed by 30 cycles (denaturation at 94 °C for 1 min, primer hybridization at 57 °C for 30 s and elongation at 72 °C for 1 min), and final extension at 72 °C for 5 min. PCR products (10 µL) were separated on 1% agarose gel containing ethidium bromide in 1 × TAE buffer (48.5-g Tris, 11.4-mL glacial acetic acid, 20 mL 0.5 M EDTA, pH 8) at 70 V for 1 h, and DNA bands were visualized under UV. DNA of a PPFM reference strain N39 [25] was taken as a positive control.

### Real-time PCR quantification of endophytic bacteria in seed, leaf, stem and root samples

One-gram surface-sterilized fresh sample was used to extract genomic DNA using FastDNA spin kit following the manufacturer’s instructions (MP Biomedicals, USA). The extracted DNA was dissolved in 100-µL TE buffer and stored at − 20 °C. The DNA concentrations were determined in a nanodrop (Klab, South Korea) by measuring absorbance at 260 nm, assuming that 1 A_260_ unit corresponds to 50 ng of DNA per microlitre. The quality of DNA was further assessed by running the DNA on a 0.8% agarose gel electrophoresis. Quantitative PCR of the total DNA sample was conducted on a StepOnePlus PCR (ABI, USA) using 1 ng.μL^−1^ of DNA template, 10 μL of 2 × SYBR green master mix (Takara) and 200 nM of primer (IDT Bangalore). The final volume of the PCR reaction mixture was made to 20 μL with PCR grade water (MP Bio, USA). Primers 799F (5′-AAC MGG ATT AGA TAC CCK-3′) and 1193R (5′-ACG TCA TCC CCA CCT TCC-3′) targeted the V5–V7 region of plant-associated bacterial communities [35]. The negative control contained no template DNA. The amplicon fragment size was about 500 bp. Thermal cycling was carried out by an initial denaturizing step at 94 °C for 4 min, followed by 40 cycles of 94 °C for 1 min, annealing temperature for 30 s and 72 °C for 45 s, and final extension was carried out at 72 °C for 5 min. Fluorescence was measured during each cycle of PCR. Ct value was determined from the dilution series of target DNA with defined target molecule amounts. The quality of PCR amplification products was determined by melt curve analysis with a temperature increase of 0.3 °C per cycle. The standard curve was prepared as previously described [36], and data were presented as gene copy numbers per gram of sample.

### Taxonomical identification of isolates by *rrn*-encoding gene sequencing

DNA template was prepared from cells taken from an isolated colony suspended in freshly prepared NaOH 1-M solution, lyzed at 95 °C for 10 min and stored at − 20 °C if needed. Before use, 1 µL of a 1/20 dilution in sterile DNAse-free water was added to the PCR reaction mix of 50 µL with 1 × GoTaq buffer (Promega) and 0.02 µM of each primer (IDT Bangalore; primer 27f, 5′-AGA GTT TGA TCC TGG CTC AG-3′ and primer 1492r, 5′-CCG TCA ATT CMT TTR AGT TT-3′) for 16S rRNA encoded *rrn* gene. PCR program includes an initial denaturation step of 94 °C for 4 min, 35 cycles of amplification (94 °C for 1 min, 52 °C for 30 s and 72 °C for 45 s), and final extension at 72 °C for 5 min. Amplification was performed on a PCR system (StepOnePlus, Applied Biosystem, USA). A PCR amplification product of 1.5 kb was gel-purified from a 1% agarose gel after electrophoresis using Geneaid DNA pure kit (Geneaid, Singapore) following the manufacturers’ instructions. DNA was sequenced for both strands using forward (27f) and reverse (1492r) primers in France (sequencer Genetic Analyzer (Applied Biosystems 3130 XL), assembled using CAP contig assembly tool of BioEdit ver 7.2.5), and the taxonomy assignment was based on SILVA rRNA tool assignment (https://www.arb-silva.de/).

### Plant tissue extraction

Plants were aseptically transferred in laminar air flow, and 1 g of surface-sterilized seed, root and leaf was homogenized in 10-mL sterile distilled water, centrifuged at 10,000 g for 10 min and supernatant passed through 0.2-µm syringe filter (HiMedia Inc., Mumbai, India) to remove microbial cells. Plant extracts were stored at − 20 °C and thawed before use for microbial or chemical tests [37].

### Methylotroph growth inhibition test by wheat plant extracts

The inhibition assays were carried out using aqueous plant extracts only to preserve the biological relevance of plant-derived metabolites under conditions that mimic their natural occurrence in plants. For the disk diffusion assay [38], sterile paper disk (HiMedia Inc., Mumbai, India) was soaked with 30 µL of plant filtrate and aseptically placed on the surface of NMS plate surface streaked with methylotrophs bacteria. Sterile water was used to serve as control. Zone of inhibition was measured, and diameter was recorded to demonstrate the inhibitory effect.

For liquid quantitative evaluation, 1 mL of plant filtrate was added in 250-mL Erlenmeyer flask containing 50 mL of NMS broth supplemented with 0.5% methanol and 1% inoculum of *Methylobacterium* sp. (OD 0.02 at 600 nm). The flasks were incubated at 30 °C in shaker incubator. After 7 days, the inoculum was serially diluted, and 100 µL was spread for CFU count after plating of culture on NMS plates in three replicates.

### Effect of peganine on methylotroph growth

Disk diffusion assay was used to evaluate the effect of peganine on methylotroph growth [38]. Peganine was obtained commercially (Himalaya Wellness Company, India): 250 mg of total active ingredients of the product (Vasaka) contained 2.5 mg of peganine. About 1 g of the product was dissolved in 10 mL of distilled water. The completely dissolved solution was then centrifuged for 10,000 g for 10 min. The supernatant was filter sterilized using a 0.2-µm syringe filter. Different concentrations (0.1–0.5 mg.mL^−1^) of supernatant were prepared in sterile distilled water and tested against *Methylobacterium* sp. using disk diffusion protocol. Autoclaved water in disk served as control. The plates were incubated for 7 days, and inhibition effect was measured in terms of clear zone formation around the sterile disk.

### Mass spectrometry characterization of root and leaf extract compounds

Before liquid chromatography (LC) mass spectrometry (MS) quadrupole time-of-flight (QToF) analysis, plant filtrates were dissolved in acetonitrile–water (1:1 v/v) and injected into C18 reverse phase column (Agilent technologies). The LC flow rate was maintained at 0.300 mL.min^−1^, initially 50% water and 50% acetonitrile, and then a linear gradient over 30 min to 10% water and 90% acetonitrile was used as mobile phase. MS was performed in positive ion mode scanned from m/z 100 to 1200. To identify the alkaloid molecules, the fraction was analysed using liquid chromatography-hybrid quadrupole time-of-flight mass spectrometry (LC–MS QToF) in positive ion mode with Agilent software QToF B.05.01 (B5125.3) and dissolved in acetonitrile 5% and water 95% followed by a linear gradient of 5% water and 10% acetonitrile for 30 min.

### Statistical analysis

All samples were carried out in triplicate, and statistical analysis was conducted using MS Office Excel to determine arithmetic mean and standard deviation. The results were statistically analysed by using WASP 2.0 software (https://ccari.icar.gov.in/wasp2.0/index.php) where one-way analysis of variance was performed for the separation of mean for significant difference among the treatments at the significance level (*p* < *0.05*).

## Results

### Validation of the sterilization protocol to remove epiphytes from roots, leaves and stems

The effectiveness of seed surface sterilization and its innocuity on seed germination as well as on growth of wheat plants was controlled. Before sterilization, the 1-g seed surface contained an average of 10^2^ bacterial cells. Growth of wheat plants was measured after 20 days of sowing. The method of surface sterilization was sufficient to disinfect the outer seed surface as no bacterial growth was observed after 24 h of incubation period in the control plates. The viability of each individual seeds was not affected due to surface sterilization process as 100% germination was observed after 48 h of seed incubation. As found with untreated seeds, the root length ranged between 10 and 12 cm, shoot length ranged between 15 and 20 cm and dry weight of root and shoot were in the range of 0.13 ± 0.02 g and 0.93 ± 0.03 g, respectively (Fig. 2). On the other hand, endophytes also remained vital after the process of sterilization as plants were raised from these surface-sterilized seeds, and subsequent isolation was done from other plant parts.

### Occurrence of methanol-utilizing endophytes was higher in wheat plant leaves than seeds and absent in roots

**Fig. 3:**
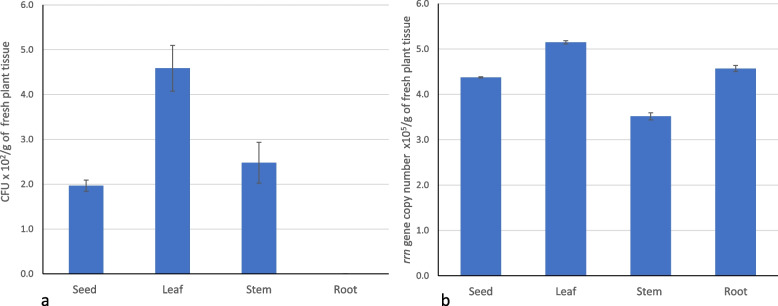
Microbial abundance in different plant parts of wheat. **a** Abundance of viable pink-pigmented facultative methylotrophs (number of CFU/g fresh weight). **b** 16S rRNA-encoding *rrn* gene quantitative PCR of the total eubacterial abundance (copy number/gram fresh weight). Each data point represents arithmetic average with standard deviation as error bar of three replicated observations and significance difference at *p* < *0.05* level

Endophyte colonies growing on methanol supplemented medium were estimated to be in the same range in CFU per gram of surface-sterilized leaf, root and stem (8 to 5 × 10^5^) and significantly less in seed (5 × 10^4^). Among the total CFU obtained, pink-pigmented endophyte CFU counts were maximum in leaf samples as compared to stem and seed, and around four times higher in leaves than in seed. No methanol-utilizing CFU were recovered from the root (Fig. 3a). Based on colony morphology and pigment observation, the bacterial isolates were selected and streaked on NMS medium supplemented with methanol.

When the abundance of the global bacterial communities was assessed using *rrn* gene-targeted quantitative PCR, the bacterial load was higher in the leaf followed by root and seed (Fig. 3b, Additional file 1: Fig. S1). Similarly, the viable counts of methanol utilizers showed that the abundance of methylotrophs was 15% higher in the endosphere of the leaf than in seed, whereas 4.3% higher in root as compared to seed. This correlates with the fact that the leaf portion of the plant bacterial load had 11% (*p* < *0.05* level) higher endophyte population than the root (Fig. 3).

### Taxonomical and phenotypical diversity of bacterial isolates from the endosphere of different plant parts

Different taxa were isolated from seed, stem and leaf samples. The only detected common species transferred from seed to stem was affiliated with *Methylobacterium* sp. Strains of *Methylobacterium* sp. were isolated from the endosphere of seed (strain SPU4), leaves (strains LPU2, LPU3, LPU5, LPU10, LPU11) and the stem of the wheat plant (strains STPU5, STPU8). *Methylobacterium* sp*.* was the only detected common species transferred from seed to stem, whereas *Pantoea* sp*.* specifically moved only towards the root region from seed. Other taxa isolated from different plant parts included species of the genera *Curtobacterium*, *Paenibacillus*, *Microbacterium*, *Pseudomonas*, *Rhizobium* and *Burkholderia* (Table 1).

**Table 1:** Occurrence of bacterial taxa isolated from the endospheres of different plant parts of wheat

*rrn*-based genus affiliation	Leaf	Root	Stem	Seed
*Methylobacterium* sp.	+	-	+	+
*Curtobacterium* sp*.*	+	-	-	-
*Burkholderia* sp.	+	-	-	-
*Paenibacillus* sp*.*	+	-	+	-
*Sphingomonas* sp*.*	+	-	-	-
*Pantoea* sp.	-	+	+	-
*Rhizobium* sp.	-	+	+	-
*Stenotrophomonas* sp.	-	+	-	-
*Microbacterium* sp.	-	+	-	+
*Enterobacteria* sp.	-	-	+	-
*Bacillus* sp.	-	-	+	-
*Pseudomonas* sp.	-	-	+	-

**Fig. 4:**
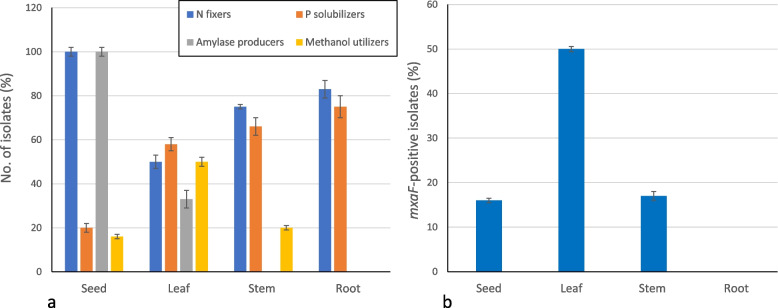
Characteristics of bacterial isolates obtained from different endosphere of plants.** a** Relative occurrence of plant growth-promoting traits of methylotrophs isolated from different parts of the wheat plants. Isolates were grown in selective media to test the properties of nitrogen fixation, P solubilization, amylase activity and methanol utilization as a carbon and energy source for growth. **b** The PCR presence of the methanol dehydrogenase marker *mxaF* gene in PPFM strains isolated confirmed the methylotrophic ability. Data represent significant difference at *p* < *0.05* level

A total of 36 selected isolates were compared for their ability to solubilize phosphate, fix nitrogen, hydrolyze starch (amylase activity) and grow using methanol or succinate as sole carbon and energy sources. Distinct biochemical profiles were observed according to the plant part endosphere from which strains were isolated. The most efficient phosphate solubilizers (zone size ~ 10 mm) were isolated from the root endosphere. Nitrogen fixers and amylase producers were found in the seed endosphere (Fig. 4a, Additional file 1: Table S2). Methanol utilizers were isolated from the phyllosphere and seed and none from roots.

Eight pink-pigmented methanol utilizers grew in liquid medium using methanol as the sole source of carbon and energy (no growth observed in the absence of methanol), unlike non-pigmented isolates. They also grew with succinate when provided as the sole carbon source, suggesting that they are PPFM. Methanol assimilation was further confirmed by the PCR amplification of the *mxaF* gene, a key marker of methanol methylotrophic oxidation. A 550 bp band was amplified in the PPFM isolates: *Methylobacterium* strains LPU5, LPU11, SPU4, LPU2, LPU3, LPU10, STPU5 and STPU8. As a conclusion, PPFM were not isolated from the wheat endosphere of roots but from seeds, stems and leaves, which represented 50% of identified PPFM (Fig. 4b, Additional file 1: Fig. S2).

### Plant root tissue extracts inhibited PPFM growth and contained high alkaloid content

Disk diffusion test indicated a clear halo zone around the disc containing root extracts, which demonstrates an inhibiting activity against PPFM. No inhibition zone was observed around disks containing extracts of the leaf, stem and seed, as well as controls without plant extracts (Additional file 1: Fig. S3a, b, e). On the other hand, root extracts in liquid broth showed maximum inhibition against the growth of PPFM strain (Additional file 1: Fig. S3f).

Using LC–MS QToF analysis, more chemical metabolites were detected in root extracts compared to shoot extracts. A total of 28 compounds had peak heights above 1%. The most abundant compound was a naturally occurring alkaloid detected in root extracts, peganine (*RT* 3.682 min, peak height 15.13 µV), identified with a 16.6% higher peak than in shoot extracts. Peganine is naturally produced by plants such as *Vasaka adhathoda* and possesses medicinal properties [39]. Other abundant identified metabolites in shoot extracts included the purine base hypoxanthine (*RT* 1.98 min, peak height 7.47 µV), methyl N-methyl anthranilate (*RT* 4.251 min, peak height 4.32 µV), which acts as an anti-herbivory defensive volatile produced by several plants [40], and the phytohormone indoleacrylic acid (*RT* 6.403 min, peak height 3.30 µV) [41] (Additional file 1: Fig. S4). Note that the use of acetonitrile–water mixtures was confined solely to LC–MS analyses, where it was required for chromatographic separation and ionization. As acetonitrile was never present in the growth inhibition assays, the observed effects can be confidently attributed to endogenous metabolites such as peganine, rather than to solvent-related artefacts.

### Effect of peganine on the growth of *Methylobacterium* strain

To elucidate the mechanism of differential partitioning of methylotrophs in different plant parts in relation to the occurrence of peganine, a follow-up experiment was carried out by testing different concentrations of commercially available peganine on the growth of *Methylobacterium* sp. Disk diffusion assay indicated that the inhibition zone diameter was proportional to the amount of peganine present in the disk (an inhibition zone of 5.5 mm corresponded to 0.5 mg of peganine) (Additional file 1: Fig. S5). In a control without peganine supplementation, no inhibition was observed. This clearly suggests that peganine directly inhibits the growth of *Methylobacterium* in a concentration-dependent manner.

## Discussion

In the wheat seed endosphere, the abundance of bacteria detected was around 2 × 10^2^ CFU/g of dry weight, which was lower than other studied endosphere parts (root, stem and leaf). This seems a general pattern in plant endosphere, regardless of applied methods, with proportion of bacteria in seeds up to 100-fold less than in other endosphere plant parts, as previously observed [12]. On the other hand, the bacterial population densities in the phylloplane (plant aerial portion like stem and leaf) ranged between 60 × 10^3^ and 85 × 10^3^ in our study, which is similar to previously reported 10^3^–10^4^ CFU of bacteria per gram of fresh weight under natural conditions [10]. Within the bacterial endophytes in wheat, we detected PPFM as the dominant taxa which is consistent with other findings conducted on *Brassica* and *Perilla* seeds [18, 28].

In our wheat study, the PPFM distribution varied greatly between different parts of the plant endosphere: from undetected in roots, low proportion in seeds and the highest in leaves. The case of a seed-transmitted uncultivated *Methylobacterium* to the shoot and root endospheric microbiome of all 16 juvenile plant species tested, including wheat, was previously reported for particular taxa (16 s rRNA partial sequence BacOTU28) [42] for which its ability to produce pigments remains unknown, as not all *Methylobacterium* are PPFM. Our results suggest a bifurcation of seed PPFM between the endosphere of root and shoot, and that a larger proportion had diverted into the shoot region. The fact that PPFM were more abundant in the shoot endosphere and absent in the root endosphere suggests that a potential selective mechanism may favour PPFM transfer towards shoot from seed. The propensity of methanol utilizers such as PPFM to inhabit such tissue was high as methanol is a by-product emitted during cell wall synthesis of the leaf [43]. The presence of methanol-sensing chemoreceptor MtpA-dependent chemotaxis and flagellin protein FliC-dependent motility in PPFM facilitates the transfer of PPFM into a methanol-emitting environment such as the phyllosphere [44]. Unlike *Methylobacterium*, *Pantoea* is a taxon reported to be highly abundant in the different parts of the endosphere of many plants, including wheat [42, 45]. Seed endophyte *Pantoea*-labelled strains were recovered from every tested part of wheat seedlings [4], which confirms that *Pantoea* endophytes in seed do not adopt a bifurcation strategy as observed with PPFM in our study.

Endophytes that inhabit different plant parts will adjust their diet to variable intracellular space content in carbohydrates, amino acids and inorganic nutrients available in each plant part [46] so that the microbiome in plant parts depends on the metabolic abilities and phenotypic traits of the endophytes [47]. Upon biochemical characterization of isolates, more amylase producers were observed inside the seed. As the seed endosperm has 80% of carbohydrate in form of starch, amylase activity is a beneficial microbial trait to promote its central metabolism [48]. Maximum nitrogen fixers were isolated from wheat seed followed by root, suggesting that in our study most of the N fixers are diverted to root regions, as described previously [49]. Enrichment of phosphate solubilizers in wheat plant root suggests that endophytes have an important role in phosphorous acquisition in root and are therefore diverted in this region. A study on poplar trees found that phosphate taken up by plant roots can be converted to insoluble form within the plants, and therefore, endophytic bacteria are advantageous to plants for releasing soluble phosphate [50]. Besides central metabolism, endophytes may produce cell wall-degrading enzymes to gain entry in other plant microcompartments [51, 52]. Plants can also control their endophyte communities by producing inhibitory compounds, whose presence can differ between parts of the plants.

In the present investigation, it was observed that PPFMs exhibited limited migration from seeds towards the root tissues, whereas their abundance was comparatively higher in the shoot compartments. This distinct colonization pattern may be attributed to two plausible mechanisms. The first relates to differences in the metabolic profiles of shoot and root tissues. Metabolite profiling revealed that the alkaloid peganine content was significantly higher in root extracts as compared to shoot extract. Peganine belongs to quinozoline alkaloidal group produced from plants [39, 53]. Wheat root extracts in our study contained peganine at a concentration of approximately 0.5 mg.mL^−1^, a level sufficient to inhibit the growth of *Methylobacterium* sp., as corroborated with the in vitro inhibitory effect of peganine on the growth of methylotrophs. Notably, peganine is well documented for its potent antibacterial activity against both Gram-positive and Gram-negative bacteria, including *Micrococcus luteus*, *Staphylococcus aureus*, and *Pseudomonas aeruginosa*, with reported minimum inhibitory concentrations ranging from 0.03 to 2 mg.mL^−1^ [54]. However, no MIC studies have thus far been conducted to specifically evaluate the effect of peganine on PPFM, underscoring a critical gap in current knowledge of plant–microbe interactions. Peganine, a secondary metabolite characterized for its antibacterial and cytotoxic properties, has been identified in plants such as *Adhatoda vasica* and *Peganum harmala* [54, 55], yet its role in shaping plant-associated microbial communities remains underexplored. The second explanation for the reduced root colonization by PPFMs may be associated with methanol metabolism. Aerobic methanol oxidation has been reported to be less prevalent among rhizospheric bacterial isolates, which may consequently limit the establishment and persistence of PPFMs in root tissues [56]. Collectively, these findings suggest that host-derived metabolites play a pivotal role in governing PPFM colonization patterns, thereby modulating plant–microbe associations.

## Conclusions

**Fig. 5:**
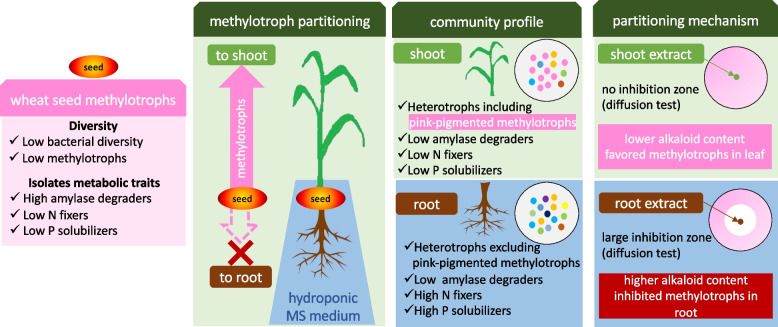
Schematic illustration of selective partitioning of methylotrophs (methanol utilizers) in root and shoot parts of the wheat plant. Methylotrophs in seeds were less in abundance. These organisms exhibited high amylase and low N-fixing and P-solubilizing properties. Methanol-utilizing methylotrophs were more abundant in shoot than root. Methanol utilizers inhabiting in shoot exhibited lower amylase and N-fixing and P-solubilizing attributes. No pink-pigmented methylotrophs were isolated from root tissue. Isolates of the root exhibited lower amylase and higher N-fixing and P-solubilizing attributes. Plant extract LC–MS analysis indicated that root tissue had higher alkaloid content than shoot tissue. Lower concentration of alkaloid in shoot may favour methylotrophs, while higher concentration of alkaloid in root restricted proliferation of PPFM

PPFM are methanol-utilizing organisms that seem to be selectively transferred from the wheat endosphere of seed to colonize the root and aerial parts of the plant. However, the PPFM were differentially distributed between shoot and root, with the absence of recoverable PPFM isolates in roots. We also found that the root content of peganine, an alkaloid metabolite, was high in 3-week-old wheat plants, and that peganine alone was sufficient to inhibit the growth of PPFM in pure cultures. Therefore, we hypothesize that *in planta*, higher peganine content combined with lower availability of methanol may limit PPFM thriving and subsequently limit colonization in the root. Figure 5 illustrates the observed preferential PPFM migration from seeds towards shoot tissues and highlights the potential mechanistic basis of PPFM spatial distribution within plant tissues. Future research is needed to better correlate methylotroph partitioning mechanisms and plant fitness under abiotic stresses.

## Supplementary Information

Additional file 1. Fig. S1– Gene *rrn* quantitative PCR standardization. Fig. S2 – PCR detection of a methanol dehydrogenase marker gene in PPFM strains. Fig. S3 – Effect of plant tissue extract on PPFM strain. Fig. S4 – LCMS QTOF analysis of root and shoot. Fig. S5 – Effect of peganine on methylotrophic strain growth. Table S1 – Detailed wheat variety PUSA HI 1605 information. Table S2 – Biochemical characterization of isolates.

## Data Availability

The sequences generated of the present study are available in the NCBI repository, available through the web link (https://www.ncbi.nlm.nih.gov/nuccore/?term=PQ112322:PQ112357%5baccn).

## Abbreviations

PPFM: Pink-pigmented facultative methylotrophs
NMS: Nitrate mineral salts
BP: Base pair
