# Supplementary material for: Differential partitioning of seed-inhabiting methylotrophs in the endosphere of wheat plants
Source: BMC Biol. 2025 Dec 12;24:13. doi: 10.1186/s12915-025-02473-8 (PMC12817747; doi:10.1186/s12915-025-02473-8)
Supplement: Supplementary file 1 — Additional file 1. Fig. S1– Gene rrn quantitative PCR standardization. Fig. S2 – PCR detection of a methanol dehydrogenase marker gene in PPFM strains. Fig. S3 – Effect of plant tissue extract on PPFM strain. Fig. S4 – LCMS QTOF analysis of root and shoot. Fig. S5 – Effect of peganine on methylotrophic strain growth. Table S1 – Detailed wheat variety PUSA HI 1605 information. Table S2 – Biochemical characterization of isolates. [file 12915_2025_2473_MOESM1_ESM.docx]

**Additional file 1**

|  | 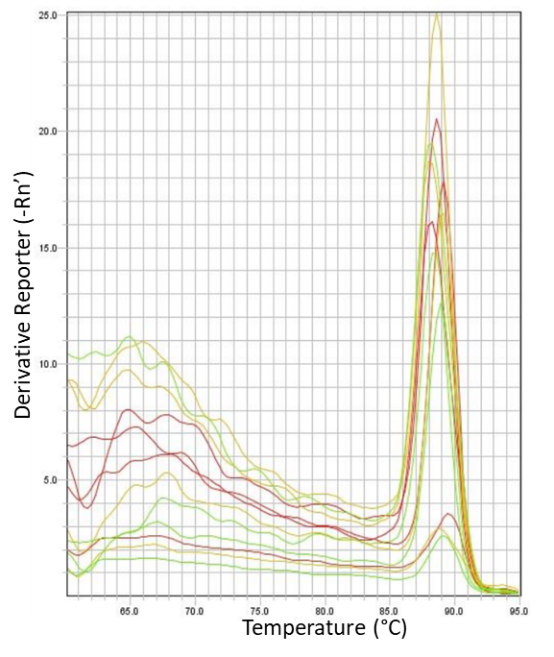 |
| --- | --- |
| **a** | **b** |

Figure S1. Gene *rrn* quantitative PCR standardization. (a) Standard plot between Ct value and gene copy number plasmid pME8309 Clone_16SrRNA. (b) Melt curve analysis of amplified product.

| **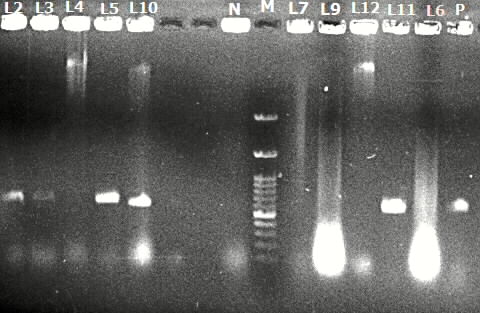** | 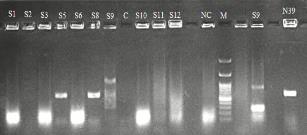 |
| --- | --- |
| **a** | **b** |
| 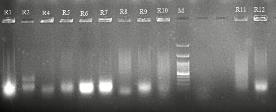 |  |
| **c** |  |

Figure S2. PCR detection of a methanol dehydrogenase marker gene in PPFM strains isolated from different wheat plant parts. Gel electrophoresis of *mxaF* gene amplicon of methylotrophs isolates grown on NMS medium with 0.5% methanol. Isolates were obtained from (a) leaf (L1-L12), (b) stem (S1-S12), (c) root (R1-R12). M-100 bp ladder; N39- positive control; N- negative control. Isolates with *mxa* genes were identified as methylotrophs.

| **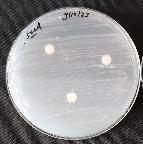** | **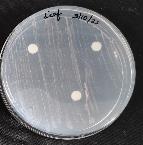** | **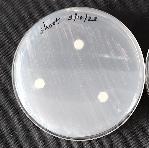** |
| --- | --- | --- |
| **a** | **b** | **c** |
| **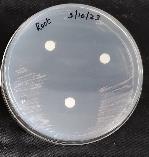** | **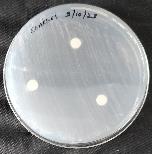** | 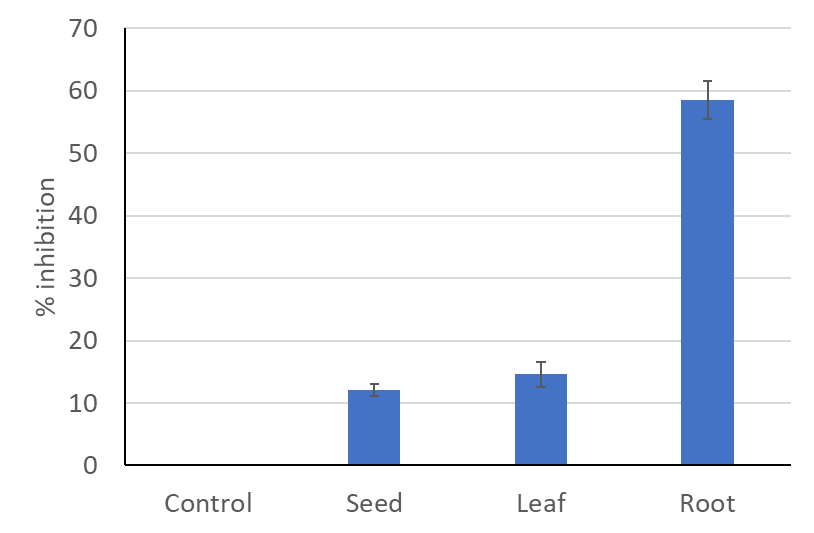 |
| **d** | **e** | **f** |

Figure S3. Effect of plant tissue extract on pink-pigmented methylotrophs growth. Effect was evaluated qualitatively by disk diffusion assay. Tissue extract from different plant parts including (a) seed, (b) leaf, (c) stem, (d) root were sterilized by passing through 0.2 micrometre syringe filter. Sterile discs impregnated with extract were placed on plates streaked densely with PPFM. (e) Control plate had discs impregnated with sterile water. Effect was observed in terms of clear zones of microbial mat around the disk. Clear zone was observed only in case of root extract. (f) Effect of 1 mL of plant tissue extract on growth of methylotrophs in 50 mL liquid broth estimated quantitatively in terms of percent inhibition of CFU obtained. X axis represents sap of different plant parts and Y axis represents %inhibition. Each data point represents arithmetic mean ± standard deviation as error bar of three replicated observations and significance difference at *p* < *0.05* level.

| 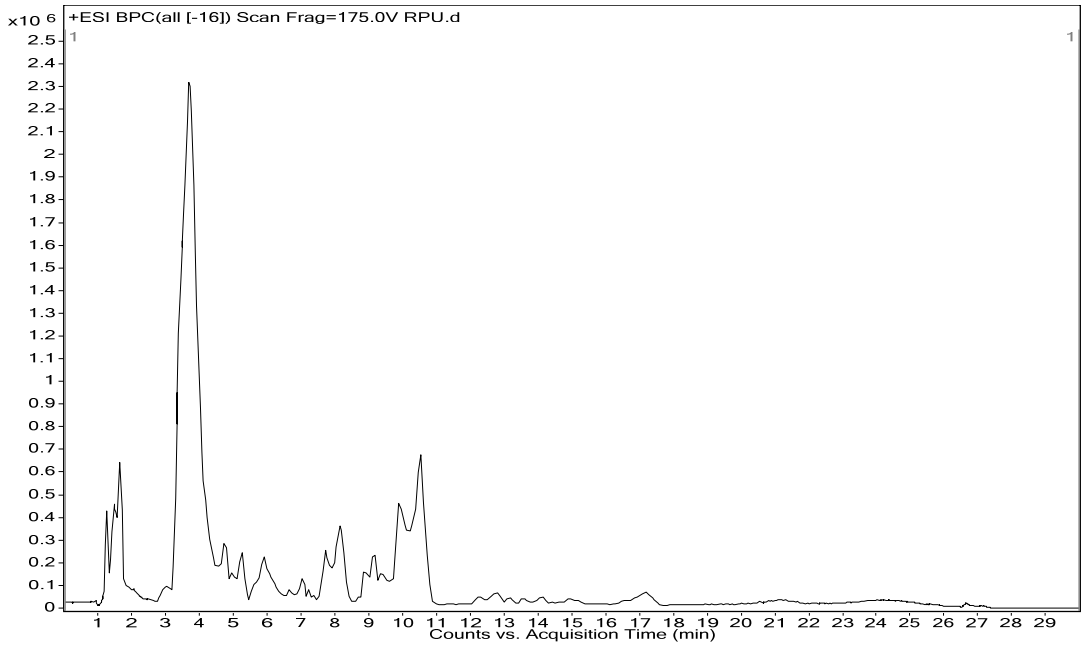 |
| --- |
| **a** |
| 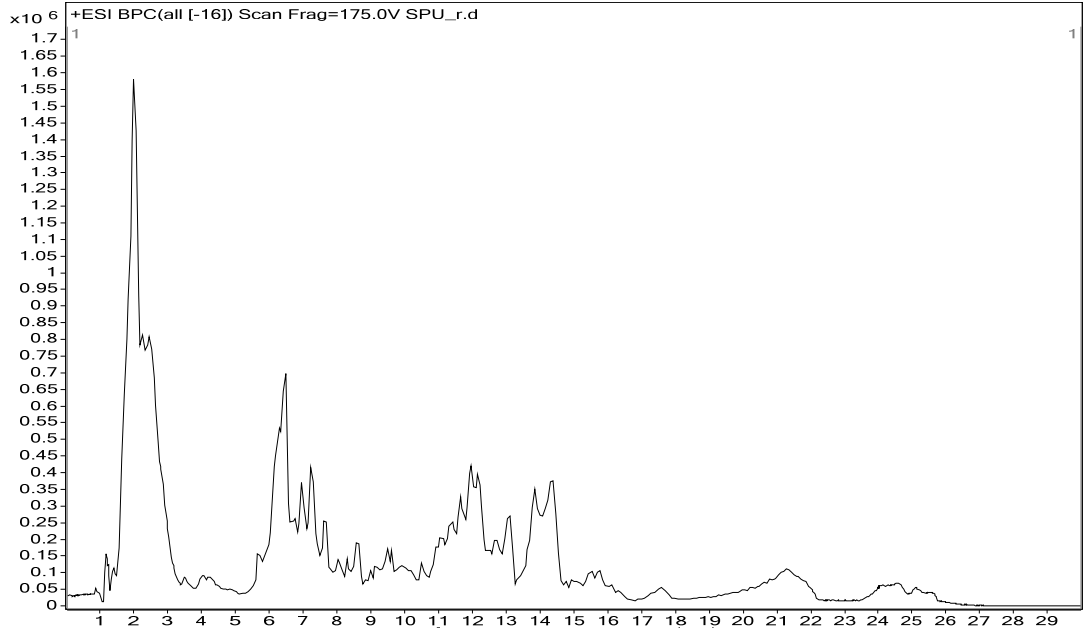 |
| **b** |

Figure S4. LCMS QTOF analysis of root (a) and shoot (b) extracts of wheat plants. X axis represents retention time in minutes and Y axis represents peak height in microvolt. Peaks represent various detected compounds.

| **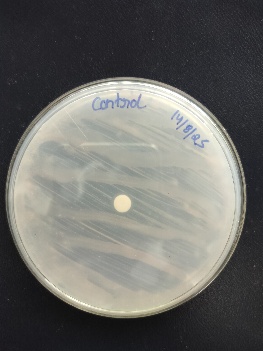** | **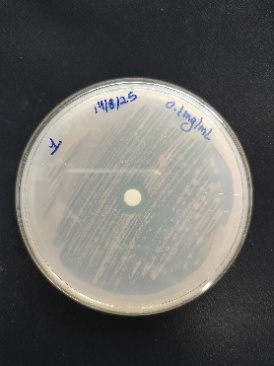** | **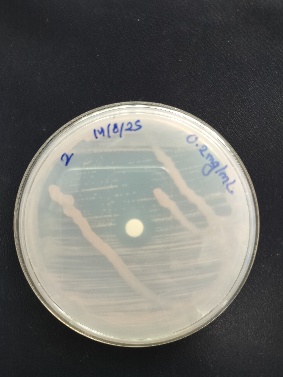** | **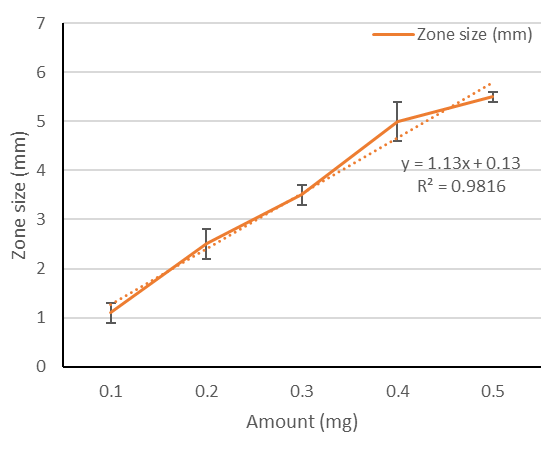** |
| --- | --- | --- | --- |
| **a** | **b** | **c** |  |
| **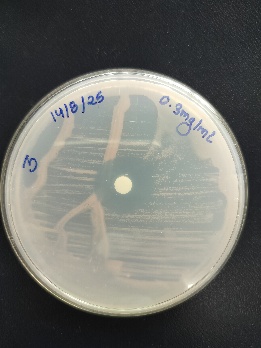** | **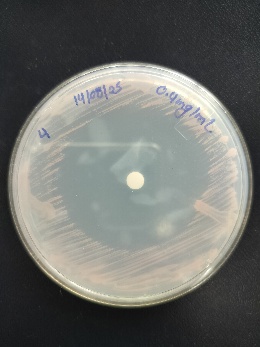** | **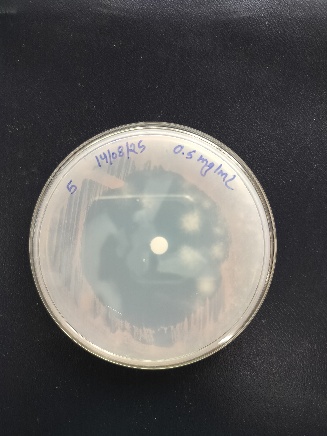** |  |
| **d** | **e** | **f** | **g** |

Figure S5. Effect of peganine on methylotroph growth by disc diffusion assay. Zones of inhibition around discs were measured for different amount of added peganine against Methylobacterium sp. (a–f from 0 to 0.5). Panel (g) represents the standard curve prepared between zone of inhibition and peganine concentration (0.1–0.5 mg). Each data point represents the mean of three replicates (n = 3), and error bars indicate standard deviation.

**Table S1.** Agronomic and quality traits of wheat variety PUSA HI 1605 employed in the present study

| **Features of wheat variety PUSA HI 1605** | **Properties of wheat variety PUSA HI 1605** |
| --- | --- |
| Yield potential | Potential high yield of 4.4 t/ha, average yield of 2.91 t/h |
| Protein content | 13% of total seed weight |
| Gluten quality | Low gluten strength |
| Utilization | Bread making |
| Lodging resistance | Moderate |
| Ripening time | 105 days |
| Winter hardiness | Moderate |
| Resistance to abiotic stresses | Drought resistance |

**Table S2.** Biochemical and molecular characterization of endophytic isolates from seed, leaf, root and stem of wheat. The biochemical attributes are presented as positive (+) or negative (-).

| **Strain name**  **& origin** | **Taxonomic identification** | **nitrogen**  **fixation** | **phosphatase activity** | **amylase activity** | ***mxaF* PCR detection** | ***rrn* gene GenBank accession no.** | |
| --- | --- | --- | --- | --- | --- | --- | --- |
| **Seed** | | | | | | |  |
| SPU1 | *Microbacterium* sp. | **+** | **-** | **+** | **-** | PQ112342.1 | |
| SPU2 | *Microbacterium* sp. | **+** | **-** | **+** | **-** | PQ112343.1 | |
| SPU3 | *Microbacterium* sp. | **+** | **-** | **+** | **-** | PQ112344.1 | |
| SPU4 | *Methylobacterium* sp. | **+** | **+** | **+** | **+** | PQ112345.1 | |
| SPU5 | *Microbacterium* sp. | **+** | **-** | **+** | **-** | PQ112346.1 | |
| SPU6 | *Microbacterium* sp. | **+** | **-** | **+** | **-** | PQ112347.1 | |
| **Leaf** | | | | | | |  |
| LPU2 | *Methylobacterium* sp. | - | - | - | **+** | PQ112322.1 | |
| LPU3 | *Methylobacterium* sp. | + | - | - | **+** | PQ112323.1 | |
| LPU4 | *Curtobacterium* sp. | - | + | + | - | PQ112324.1 | |
| LPU5 | *Methylobacterium* sp. | - | - | - | **+** | PQ112325.1 | |
| LPU6 | *Burkholderia* sp. | + | + | + | - | PQ112326.1 | |
| LPU7 | *Paenibacillus sp.* | + | - | - | - | PQ112327.1 | |
| LPU9 | *Curtobacterium* sp. | + | + | + | - | PQ112328.1 | |
| LPU10 | *Methylobacterium* sp. | - | + | - | **+** | PQ112329.1 | |
| LPU11 | *Methylobacterium* sp. | - | + | - | + | PQ112330.1 | |
| LPU12 | *Sphingomonas* sp. | + | - | + | - | PQ112331.1 | |
| **Root** | | | | | | |  |
| RPU1 | *Pantoea* sp. | + | + | - | - | PQ112332.1 | |
| RPU2 | *Rhizobium* sp. | - | - | - | - | PQ112333.1 | |
| RPU3 | *Rhizobium* sp. | + | - | - | - | PQ112334.1 | |
| RPU4 | *Strenotrophomonas* sp. | - | + | - | - | PQ112335.1 | |
| RPU5 | *Pantoea* sp. | + | + | - | - | PQ112336.1 | |
| RPU6 | *Microbacterium* sp. | + | + | - | - | PQ112337.1 | |
| RPU7 | *Pantoea* sp. | + | + | - | - | PQ112338.1 | |
| RPU8 | *Pantoea* sp. | + | + | - | - | PQ112339.1 | |
| RPU9 | *Pantoea* sp. | + | + | - | - | PQ112340.1 | |
| RPU10 | *Pantoea* sp. | + | - | - | - | PQ112341.1 | |
| **Stem** | | | | | | |  |
| STPU1 | *Microbacterium* sp. | + | + | - | - | PQ112348.1 | |
| STPU2 | *Rhizobium* sp. | + | + | - | - | PQ112349.1 | |
| STPU3 | *Enterobacteria* sp. | + | + | - | - | PQ112350.1 | |
| STPU4 | *Paenibacillus* sp. | - | - | - | - | PQ112351.1 | |
| STPU5 | *Methyl bacterium* sp. | - | - | - | **+** | PQ112352.1 | |
| STPU6 | *Pantoea* sp. | + | + | - | - | PQ112353.1 | |
| STPU8 | *Methylobacterium* sp. | + | - | - | **+** | PQ112354.1 | |
| STPU9 | *Bacillus* sp. | - | - | - | - | PQ112355.1 | |
| STPU10 | *Enterobacteria* sp. | + | + | - | - | PQ112356.1 | |
| STPU11 | *Pseudomonas* sp. | + | + | - | - | PQ112357.1 | |
